# A Smartphone Attention Bias App for Individuals With Addictive Disorders: Feasibility and Acceptability Study

**DOI:** 10.2196/15465

**Published:** 2019-09-12

**Authors:** Melvyn Zhang, Jiangbo Ying, Syidda B Amron, Zaakira Mahreen, Guo Song, Daniel S S Fung, Helen Elizabeth Smith

**Affiliations:** 1 National Addiction Management Service Institute of Mental Health Singapore Singapore; 2 Department of Developmental Psychiatry Institute of Mental Health Singapore Singapore; 3 Family Medicine and Primary Care Lee Kong Chian School of Medicine Nanyang Technological University Singapore Singapore

**Keywords:** attention bias, cognitive bias, psychiatry, substance abuse, alcohol abuse, opioid abuse, cannabis abuse, addiction, digital health, mhealth

## Abstract

**Background:**

Conventional psychology therapies are unable to address automatic biases that result in individuals relapsing into their substance use disorder. Advances in experimental psychology have led to a better understanding of attention and approach biases and methods to modify these biases. Several studies have demonstrated the effectiveness of bias modification among clinical cohorts. The advances in mobile health technologies have allowed remote delivery of these interventions. To date, there is a lack of studies examining bias modification in a substance-using non-Western sample.

**Objective:**

This study was designed to determine the feasibility of an attention bias modification intervention and an attention bias modification smartphone app for the reduction of attention biases among treatment-seeking individuals. The secondary aim is to determine the acceptability of the intervention.

**Methods:**

A feasibility study was conducted among inpatients who were in their rehabilitation phase at the National Addictions Management Service. Participants were to complete a set of baseline questionnaires, and on each day that they are in the study, undertake an attention bias assessment and modification task while completing a visual analogue scale to assess their craving. Feasibility was determined by the acceptance rate of participation and participants’ adherence to the interventions. Acceptability was assessed by a perception questionnaire. Descriptive statistical analyses were performed using SPSS version 22. A thematic analysis approach was used in the qualitative synthesis of users’ perceptions.

**Results:**

Of the 40 participants invited to participate in the feasibility study, 10 declined, yielding an acceptance rate of 75%. Of the recruited participants, 6 participants were diagnosed with alcohol dependence; 17, with opioid dependence; 2, with cannabis dependence; and 5, with stimulant dependence. In addition, of the final 30 participants, 11 (37%) failed to complete all the planned interventions and 22 (73%) completed the perspective questionnaires; of these 22 participants, 100% rated the app as extremely and very easy, 77% rated it as extremely or very interactive, 54% rated it as extremely or very motivating, and 33% reported a change in their confidence levels.

**Conclusions:**

Our results highlight the feasibility of recruiting participants to undertake attention bias modification interventions. Participants generally accept use of a mobile version of such an intervention. Nevertheless, our acceptability data indicate that there could be improvements in the existing app, and a participatory design approach might be helpful in its future conceptualization.

**International Registered Report Identifier (IRRID):**

RR2-10.2196/11822

## Introduction

According to the United Nations Office on Drugs and Crime, substances like cannabis, opioids, and stimulants are commonly abused [1]. Substance abuse and substance dependence are associated with significant morbidity and mortality. An estimated 190,000 deaths were attributed to substance use in 2015 [1]. The recent statistics from the World Health Organization also highlighted the high prevalence of alcohol use [2], particularly in higher-income countries. In Singapore, the most recent study found that the prevalence of alcohol use disorders was 0.19% and 1.40% among women and men, respectively, and the prevalence of drug use disorders was 0.07% and 0.28% among women and men [3]. Given the prevalence of these disorders, there is clearly a need for effective interventions. Conventionally, treatment and interventions of addictive disorders involve a combination of medications and psychological therapies. For psychological therapies to help individuals achieve abstinence, frequently used therapies include cognitive behavioral therapy, cue-exposure therapy, contingency management, and mindfulness-based relapse prevention. Cognitive behavioral therapy has had an immediate effect size of 0.45 [4]; 40%-50% of the individuals relapsed within a year and 70% relapsed within 3 years [4]. This may be because such therapies mainly address the cognitive control processes, but fail to address the underlying unconscious automatic processes that contribute to an individual’s lapse and relapse.

Among individuals with addictive disorders, there are two common biases: attentional bias and approach bias. Attentional biases are automatic, unconscious processes that result in the preferential allocation of attention toward substance-related stimuli [5,6], and approach biases are the automated tendencies for individuals to reach out and approach substance stimuli [7]. These biases have been well-studied with a theoretical underpinning based on the dual-process model, which posits that the chronic administration of the substance leads to enhanced automatic processing of the substance-related cue, with a corresponding inhibition in the normal cognitive control processes [8]. Interventions to modify bias have been extensively evaluated. Ziaee et al [9] reported that the introduction of bias modification reduced attentional biases among individuals maintained on methadone as well as their cravings to use, the dose of methadone, and the number of relapses. Several other studies that have involved participants undergoing detoxification on an addiction treatment unit [10-12] also found that bias modification was effective in reducing attentional biases. More recently, Cristea et al [13] highlighted that bias modification interventions were effective in modifying both attentional and approach biases in individuals with alcohol and tobacco use issues [13].

Although conventional bias modification interventions are confined to a laboratory setting, advances in technologies now enable remote delivery of such interventions. A recent review [14] showed that seven of eight studies reported the effectiveness of a mobile-based cognitive bias modification intervention. These studies were targeting conditions such as insomnia, social anxiety, tobacco use, and alcohol use disorders [14]. Another recent review [15] explored the utilization of technology for retraining of attentional biases in individuals with tobacco use disorder and reported that mobile delivery of bias retraining was effective. Subsequently, the effectiveness of a mobile health approach for methamphetamine use disorder [16] was evaluated, and improvements in cognitive impairments and impulsive control, but not attentional biases, were reported [16].

To date, no study has evaluated bias modification in a substance-using, treatment-seeking, non-Western cohort. Although technologies have been used for the delivery of remote bias modification intervention, the evaluation was limited to alcohol and tobacco disorders and stimulant disorders. Although a previous study reported negative findings in this regard [16], future research should seek to evaluate the effectiveness of mobile apps for modification of attentional biases, given that the training task used in that study was a newly developed task and differs significantly from conventional bias assessment and modification paradigms. In addition, according to the recommendations of the National Institute of Health Research [17], a feasibility study is crucial, as such a study seeks to determine primarily whether a study could be conducted. This is pertinent in our case, as there are no prior studies that examined bias assessment and retraining in a treatment-seeking, non-Western cohort. In addition, feasibility studies are typically limited to the evaluation of important parameters that will be crucial in the design of the main study and do not routinely evaluate the main outcome of interest [17]. Typically, a feasibility study is conducted first, before a pilot, as a pilot study. It is essentially a version of the main study that is run on a small scale to test whether the components of the main study can all work together [17].

Our study aimed to examine the feasibility of a mobile-based attention bias modification intervention among treatment-seeking individuals with alcohol or substance use disorders. If deemed feasible, this will guide further pilot and definitive randomized trials investigating the effectiveness of the mobile intervention. The objectives of the study were to determine (1) the feasibility of participants undertaking a mobile attention bias modification intervention, (2) the feasibility of the mobile intervention for reducing attention biases, and (3) the acceptability of the intervention. The specific research questions were as follows: (1) Will the mobile attention bias modification intervention be feasible and acceptable among individuals with addictive disorders? (2) Is the developed mobile intervention capable of assessing for and reducing attention biases?

## Methods

### Study Setting and Design

The target population comprised individuals admitted for inpatient medication-assisted detoxification and rehabilitation (total duration of 14 days, with 7 days in detoxification and 7 days in rehabilitation) at the National Addictions Management Service (NAMS), Institute of Mental Health, Singapore. At NAMS, the treatment is entirely voluntarily, which implies that patients and participants could self-discharge at any time. The NAMS inpatient unit has approximately 22 beds, and most of these beds are occupied by patients who are undergoing detoxification. Patients who had completed their detoxification treatment and were in the rehabilitation phase of the program were recruited. The study design is that of a feasibility study, where participants are recruited by means of convenience sampling.

### Ethics Approval

This study was approved by the National Healthcare Group’s Domain Specific Research Board (reference number: 2018/00316) on May 2, 2018.

### Recruitment and Sample Size

Patients were recruited on completion of their inpatient detoxification treatment (7 days) and at the start of day 1 of their rehabilitation treatment. Potential participants were identified by their primary psychiatrist, provided with further information by the study team, and given time to consider participation. Participants who agreed to participate completed an informed consent form, which was signed in the presence of an impartial witness, in accordance with the Human and Biomedical Act regulations. As the study was designed to assess feasibility and acceptability, power computation was not performed. Given the diversity of the disorders, the minimum recruitment target was 30 participants and the maximum was 34 participants.

### Inclusion and Exclusion Criteria

Patients were included in the study if they were aged between 21 and 65 years; diagnosed with a primary psychiatric disorder of alcohol, opioid, cannabis, stimulants, or polysubstance dependence; diagnosed with polysubstance dependence, with alcohol, opioid, cannabis, or stimulants as the main substance of use; able to read and write in English; and capable of using a smartphone or tablet device.

Patients were excluded from the study if they had a known history of cognitive impairment or dementia, a history of seizures or a prior history of withdrawal seizures, a history of migraines triggered by flashing lights, and moderate to severe comorbid psychiatric disorders based on clinical assessment.

### Measures

Baseline demographic and clinical information was collected from the participants. This included information about nationality, gender, marital status, race, religion, highest level of education, housing conditions, current substance use, method of consumption of substance, quantity of substance consumed each time, frequency of use, previous treatment history, chronic diseases (psychiatric or physical disorders), and current psychiatric medications. Participants also completed a modified Addiction Severity Index (ASI)-Lite, Severity of Drug Dependence Scale (SDS), and the Short Form (SF-12) questionnaires.

The ASI-Lite collated information for the following domains: drug and alcohol use, medical, employment/school, legal, family, and social and psychiatric aspects [18]. In our modified version, we retained only the drug and alcohol use questions. Participants were asked about their alcohol and substance use in the last 30 days, last month, and lifetime. Participants were asked whether they had used alcohol, nicotine, heroin, amphetamine-type stimulants, cannabis, other opioids, benzodiazepines and other sedatives, barbiturates, ketamine, cocaine, inhalants, hallucinogens, and new psychoactive substances. The SDS comprised five items, all of which are explicitly concerned with the psychological components of dependence [19]. A previous study [20] reported that the total severity score is highly positively correlated with the severity of dependence, as measured by the Diagnostic and Statistical Manual of Mental Disorders-IV. The SF-12 has been widely used in the assessment of the self-reported quality of life. It only covers the eight health domains from the original SF-36 [21] and has demonstrated good content and criterion validity without any evidence of any systematic biases [22].

### Intervention

Following completion of enrollment, participants were required to complete a visual-analogue scale for craving before and after the completion of each session. Members of the study team familiar with the app gave participants a 15-minute briefing on the use of the mobile app before the commencement of the assessment and intervention. The study team provided participants tablets to use the mobile attention bias modification intervention.

On day 1 of the intervention, participants were required to complete both a baseline attention bias assessment task and an attention bias modification task. They could rest for 15 minutes before they completed a reassessment of their attention bias. On the subsequent days (days 2-7) of their rehabilitation, they completed the attention bias modification task and were allowed 10 minutes of rest before retaking an attention bias assessment task. Participants were required to complete the visual-analogue scale for craving before and after completion of each bias modification task. Participants who completed three sessions were asked to complete the app perception questionnaire. Participants were expected to undertake the intervention on each day of their rehabilitation stay, except for weekends and public holidays. Participants were allowed to undertake the intervention a maximum of five times in total.

**Figure 1:**
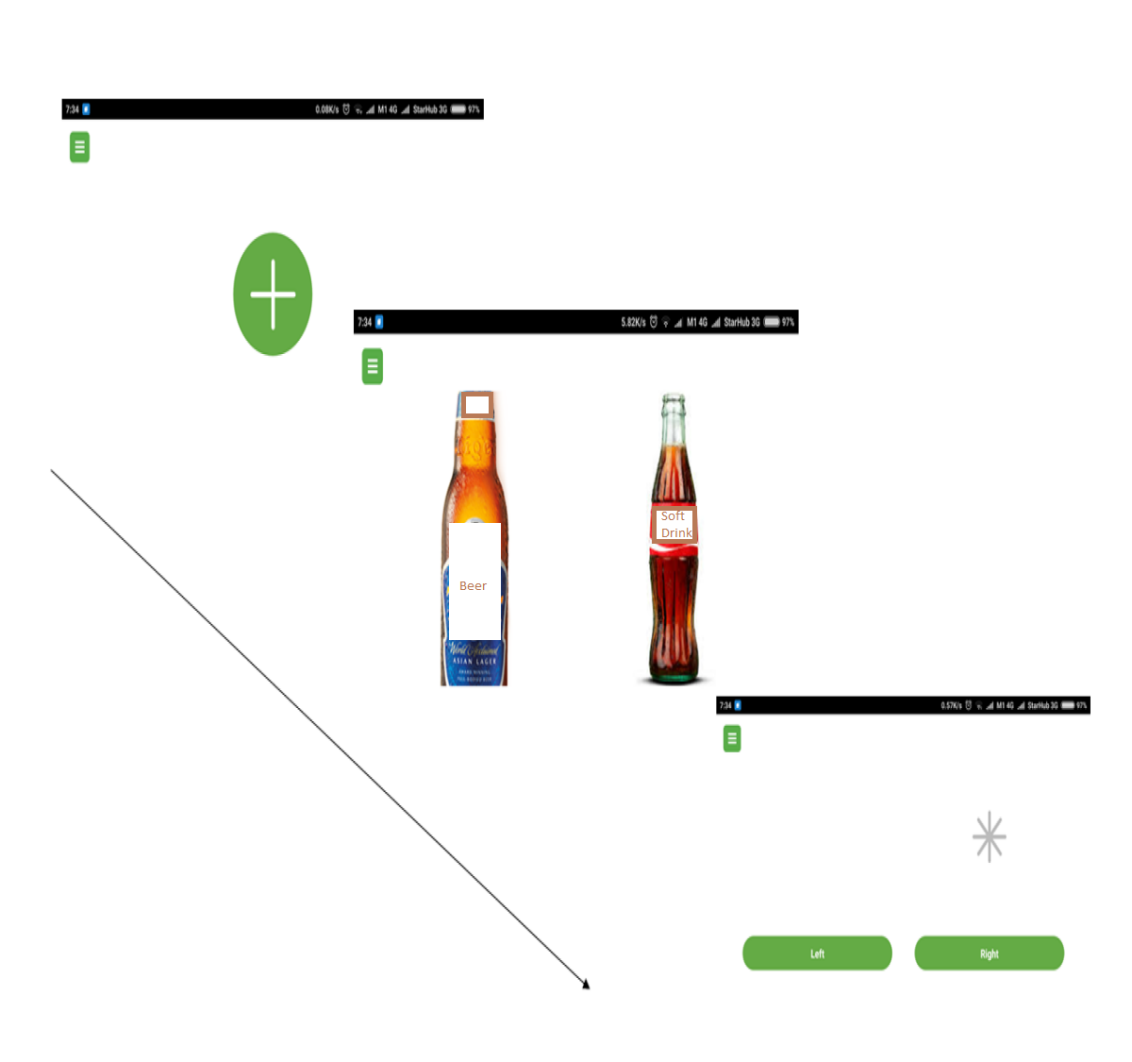
An overview of the task that participants undertake on the smartphone/tablet device.

The mobile version of the visual probe task was the same as the original visual probe task. In the attention bias assessment task, participants were required to complete a total of 200 trials (with 10 sets of images repeated 20 times). In each trial, participants were presented with a fixation cross in the center of the screen for 500 milliseconds. Subsequently, they were presented with a set of two images for another 500 milliseconds. In each set of images, one of the images was neutral but closely related to the image of the alcohol or drug (for example, an image of a man drinking from a can of beer, which was paired with an image of a man drinking from a soft drink can). Following the disappearance of the images, an asterisk replaced the position of one of the images (either on the right or left). The participants were required to indicate where the position of the asterisk was by selecting the physical on-screen buttons as fast as they could. The next set of images was presented once the participant has indicated a response (by pressing the left or right button, depending on where the asterisk was) or if the time of 2000 milliseconds had lapsed (Figure 1). In the assessment phase, 50% of the time, the asterisk replaced the neutral image and 50% of the time, the asterisk replaced the alcohol or substance image. For the intervention or bias modification task, the participant was required to take the same task as that described, but the asterisk replaced the position of the neutral image 100% of the time, enabling retraining of attentional bias. The substance images presented to participants are either a picture of the drugs, pictures of individuals using substances, or paraphernalia used for the administration of the drugs.

### Outcomes

Feasibility was the primary outcome and defined by the number of participants recruited and participants’ adherence to the intervention. The study was considered feasible if 25% of the recruitment target (of 30 participants) was met and 60% of the patients managed to adhere to the planned interventions (ie, completed all the planned interventions up until day 5 of their program).

The secondary outcome of acceptability was assessed through a perception questionnaire, which included the following questions:

1. Prior to using the app, how confident are you in managing your addiction problems? (5-point Likert scale, ranging from not at all to extremely)
2. How easy was it to use the app? (5-point Likert scale, ranging from not at all to extremely)
3. How interactive was the app? (5-point Likert scale, ranging from not at all to extremely)
4. Do you feel motivated to continue using the app? (5-point Likert scale, ranging from not at all to extremely)
5. Do the images in the app remind you of your substance use? (5-point Likert scale ranging from not at all to extremely)
6. After using the app, how confident are you in managing your addiction problem? (5-point Likert scale, ranging from not at all to extremely)

For questions 2, 3, and 4, respondents were also asked to provide free-text comments.

Acceptability was predefined as a willingness to use the app daily, and if at least 30% of the participants rated ease of use, interactivity, motivation, and reality (questions 2, 3, 4, and 5) positively (either very or extremely on the 5-point Likert scale), and if at least 30% of the participants perceived there to be a change in their confidence level after receiving three sessions of the intervention task (questions 1 and 6). The app was also deemed acceptable by the absence of any severe adverse events (such as intense cravings leading to premature discharge from the inpatient program).

**Figure 2:**
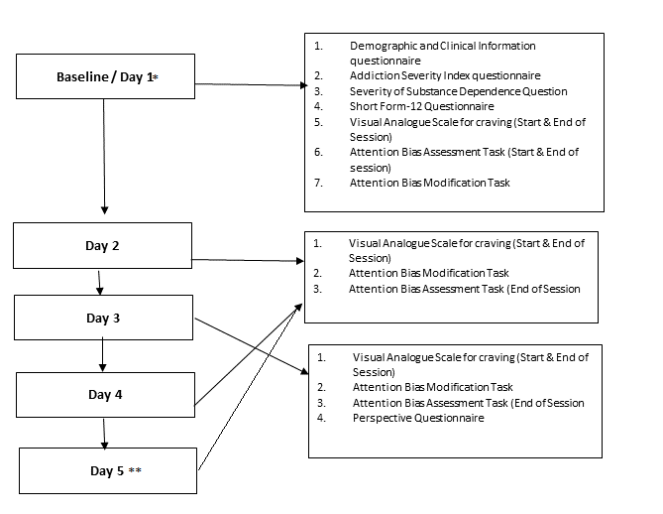
Overview of the outcomes measures that participants need to complete for each session. *Attention bias modification assessment task will be completed twice on the first day. The first assessment will provide information pertaining to the baseline attentional biases. The second assessment will assess for the change in attentional biases following the first intervention. **Participants will undertake a maximum of 5 sessions, taking into consideration that the study will not be conducted on weekends.

Figure 2 provides an overview of the task that participants undertake each day they are in the study.

### Data Management and Monitoring

All participants were allocated a subject number upon recruitment, and no participant-related identifiers were captured on the hard-copy forms. These forms, together with the questionnaires, were stored in secured, locked cabinets in a restricted area. The electronic data from the smartphone app was automatically synchronized onto a secured, password-protected cloud database. The main investigator backed-up a copy of the electronic data records onto a local secured computer daily. The principal investigator and the research assistants took the responsibility of coding the data from the hard-copy forms. An independent coinvestigator routinely checked the data entry for accuracy, ensuring that the translation of scores from the hard-copy forms to the electronic form was free of errors. All records will be kept securely for at least 6 years after completion of the study.

### Statistical Analyses

Data collated was analyzed using SPSS (version 22. IBM Corp, Armonk, NY). Baseline demographic information of the subjects was summarized using descriptive statistics, including means and SD. The presence of attentional biases was determined based on the mean reaction times taken to respond to the position of the probes that replace drug or neutral stimuli. The formula used for the computation of attentional biases was (∑T_1_/n_1_) – (∑T_0_/n_0_), where T_1_ refers to the time for probes that replaced the neutral stimulus, n_1_ refers to the number of trials for probes that replaced the neutral stimulus, T_0_ refers to the time for probes that replaced the substance stimulus, and N_0_ refers to the number of trials for probes that replaced the substance stimulus.

### Qualitative Analysis of Acceptability Data

Patients’ perspective and feedback were collated by means of the perspective questions. Two separate independent researchers coded their verbatim, handwritten comments using NVivo, version 12.0 (QSR International, London, United Kingdom). Similar codes were grouped together and further analyzed, giving rise to higher-order themes.

## Results

### Feasibility of Recruitment and Adherence

Of the 40 participants invited to participate in the feasibility study, 10 declined, yielding an acceptance rate of 75%. Of the recruited participants, 6 participants were diagnosed with alcohol dependence; 17, with opioid dependence; 2, with cannabis dependence; and 5, with stimulant dependence. In addition, 11 participants of the 30 participants failed to complete all the planned interventions. The adherence rate was thus 63%. For 10 participants, discontinuation was linked with them electing for premature discharge from the ward, and another participant withdrew from the study after the initial intervention. Table 1 provides an overview of the baseline demographic characteristics of the 30 participants recruited.

The mean age of the participants with alcohol and opioid dependence was 43.7 (SD 11.6) years and 47.9 (SD 11.8) years, respectively, and that for participants with stimulant dependence and cannabis dependence was 37.6 (SD 7.0) and 58.0 (SD 1.4), respectively. Most of the participants were Singaporean (90%), and most were of male gender (86%). In addition, 53% had a secondary school education, 76% were unemployed, and 20% of the participants reported being homeless. Furthermore, 50% of the participants with alcohol dependence and 50% of the participants with cannabis dependence had comorbid medical conditions. Moreover, 60% of the participants with stimulant dependence reported having an underlying psychiatric disorder. Participants with alcohol dependence had a mean score of 11.2 (SD 1.9) on the severity of substance dependence questionnaire; those with opioid dependence, stimulant dependence, and cannabis dependence had mean scores of 11.7 (SD 2.2), 9.0 (SD 5.7), and 8.8 (SD 4.5), respectively. These scores demonstrated that participants sampled had a psychological dependence on the substances they were using. The physical health and mental health composite scores were lower for individuals with alcohol use disorders as compared to those with the other disorders.

Table 2 provides the mean attention bias scores for each participant across the trials. Based on the protocol, participants were expected to complete a total of five training sessions. However, not all participants have completed a total of five sessions, as some participants had a public holiday during their stay. Of the 30 participants, 14 participants had positive attentional biases at baseline, whereas the other 16 participants did not have any underlying baseline attentional biases. For those with baseline attentional biases, there was a general decrease in the attention bias scores from baseline to the end of the planned intervention trials. The changes in the scores ranged from 12.0 to 409.5 milliseconds, comparing the final attention bias scores (upon the completion of the intervention) with the baseline scores (at the start of the intervention).

**Table 1:** Baseline demographic characteristics of participants (n=30).

Demographic characteristics		Alcohol dependence (n=6)	Opioid dependence (n=17)	Cannabis dependence (n=2)	Stimulant dependence (n=5)
**Age (years), mean (SD)**		43.7 (11.64)	47.9 (11.8)	58.0 (1.4)	37.6 (7.0)
**Nationality, n (%)**					
	Singaporean	4 (66.7)	16 (94.1)	2 (100)	5 (100)
	Others	2 (33.3)	1 (5.9)	0 (0)	0 (0)
**Gender, n (%)**					
	Male	4 (66.7)	15 (88.2)	2 (100)	5 (100)
	Female	2 (33.3)	2 (11.8)	0 (0)	0 (0)
**Race, n (%)**					
	Chinese	2 (33.3)	2 (13.3)	0 (0)	3 (60.0)
	Malay	1 (16.7)	11 (64.7)	0 (0)	0 (0)
	Indian	3 (50.0)	3 (17.6)	1 (50.0)	2 (40.0)
	Others	0 (0)	1 (5.9)	1 (50.0)	0 (0)
**Religion, n (%)**					
	Christianity	2 (33.3)	4 (23.5)	0 (0)	2 (40)
	Hinduism	1 (16.7)	0 (0)	0 (0)	2 (40)
	Muslim	2 (33.3)	12 (70.6)	2 (100)	0 (0)
	Others	1 (16.7)	1 (5.9)	0 (0)	1 (20)
**Education, n (%)**					
	Primary education	2 (33.3)	3 (17.6)	1 (50.0)	1 (20.0)
	Secondary education	2 (33.3)	11 (64.7)	1 (50.0)	2 (40.0)
	Junior college/polytechnic/technical studies	1 (16.7)	3 (17.6)	0 (0)	1 (20.0)
	Undergraduate studies	1 (16.7)	0 (0)	0 (0)	1 (20.0)
**Employment, n (%)**					
	Unemployed	6 (100)	12 (70.6)	1 (50.00)	4 (80.0)
	Part-time employment	0 (0)	1 (5.9)	1 (50.0)	1 (20.0)
	Full-time employment	0 (0)	4 (23.5)	0 (0)	0 (0)
**Housing, n (%)**					
	Homeless	1 (16.7)	3 (17.6)	0 (0)	2 (40.0)
	1 room	3 (50.0)	5 (29.4)	0 (0)	0 (0)
	2 rooms	0 (0)	2 (11.8)	0 (0)	0 (0)
	3 rooms	1 (16.7)	1 (5.9)	0 (0)	1 (20)
	4 rooms	1 (16.7)	3 (17.6)	1 (50.0)	2 (40.0)
	5 rooms	0 (0)	2 (11.8)	0 (0)	0 (0)
	Others	0 (0)	1 (5.9)	1 (50.0)	0 (0)
Presence of other chronic diseases, n (%)		3 (50.0)	7 (41.2)	1 (50.0)	1 (20.0)
Presence of psychiatric disorder, n (%)		1 (16.7)	0 (0)	0 (0)	2 (40)
Severity of Substance Dependence scores, mean (SD)		11.2 (1.9)	11.7 (2.2)	9.0 (5.7)	8.8 (4.5)
**Short Form-12 questionnaire scores,** **mean (SD)**					
	Physical health composite scores	40.5 (24.5)	54.7 (21.6)	82.0 (15.6)	70.2 (21.2)
	Mental health composite scores	29.3 (11.1)	41.0 (17.3)	52.0 (0)	39.2 (24.3)
	Total scores	33.7 (9.2)	46.5 (16.3)	64.5 (6.4)	51.4 (15.2)

**Table 2:** Change in attentional bias scores.

Participant	Drug	Baseline		Session 1		Session 2		Session 3		Session 4		Session 5		Overall change in attentional bias
		Attentional bias	Task ratio (neutral: drug)	Attentional bias	Task ratio (neutral: drug)	Attentional bias	Task ratio (neutral: drug)	Attentional bias	Task ratio (neutral: drug)	Attentional bias	Task ratio (neutral: drug)	Attentional bias	Task ratio (neutral: drug)	
1	Stimulants	30.3	96:100	70.6	99:101	36.3	98:102	9.3	98:102	–23.6	96:98	13.3	99:99	17
2^a^	Stimulants	–22.4	103:97	–23.4	97:103	–11.7	95:105	N/A^b^	N/A	N/A	N/A	N/A	N/A	10.7 (increased)
3	Stimulants	6.7	100:100	–3.6	98:101	–28.9	99:99	–11.3	100:99	4.1	100:99	–7.3	100:99	14
4^c^	Opioid	32.1	96:93	28.7	99:100	12.2	99:100	31.2	100:99	20.1	100:99	N/A	N/A	12
5^c^	Alcohol	91.2	97:98	–23.3	100:99	–37.4	100:99	–5.3	99:100	–33.2	99:100	N/A	N/A	124.4
6^c^	Opioid	98.9	28:116	33.4	60:46:00	14.5	71:66	–9.7	88:86	–31.5	86:84	N/A	N/A	130.4
7	Stimulants	–30.5	100:100	–13.5	100:99	–23.4	98:99	–27.7	100:99	–28.2	98:99	–7.6	96:99	22.9 (increased)
8^a^	Opioids	58.7	36:56:00	85.1	40:57:00	N/A	N/A	N/A	N/A	N/A	N/A	N/A	N/A	26.4 (increased)
9^a^	Opioids	25.8	97:97	13.5	96:98	N/A	N/A	N/A	N/A	N/A	N/A	N/A	N/A	12.3
10	Cannabis	–9.9	97:99	–20.7	60:40:00	–54.6	93:90	–14.1	96:95	–22.9	99:100	–48.4	100:99	38.5
11	Opioids	–30.9	33:66	2.4	97:97	–15.4	99:99	–7.4	97:99	14.2	99:99	7.3	98:99	38.2 (increased)
12^a^	Opioids	0.7	99:97	34.8	100:99	–15.3	98:99	–52.4	100:98	N/A	N/A	N/A	N/A	53.1
13	Alcohol^d^	–20.9	99:98	–12.3	100:98	N/A	N/A	N/A	N/A	–75.8	90:92	–48.6	99:100	27.7
14^a^	Alcohol	397.7	26:94	44.2	97:98	45.3	99:99	–32.0	98:100	–11.7	99:100			409.4
15	Cannabis	–7.7	100:96	–40.5	100:99	8.6	99:100	–33.6	99:99	–31.8	99:100	–50.3	100:99	42.6
16^a^	Opioids	–27.4	93:96	N/A^d^	N/A	N/A	N/A	N/A	N/A	N/A	N/A	N/A	N/A	N/A
17	Opioids	–42.5	49:49:00	–64.8	75:71	63.4	63:61	8.9	73:78	–15.5	78:82	26.7	96:92	69.2 (increased)
18	Opioids	27.9	92:92	–17.8	99:96	3.2	100:99	–22.6	97:97	–104.6	99:99	–9.2	97:95	37.1
19^a^	Opioids	3.8	99:96	35.1	99:98	13.7	99:98	32.4	100:98	N/A	N/A	N/A	N/A	28.6 (increased)
20	Opioids	10.1	99:98	9.4	100:98	105.2	99:98	54.1	100:99	–1.7	100:99	20.3	99:100	10.2 (increased)
21	Alcohol	224.5	79:54:00	61.5	98:97	73.3	99:100	176.7	94:97	130.3	98:100	107	99:97	117.4
22	Opioids	–52.9	100:99	10.9	99:100	5.6	98:98	39.1	99:99	36.5	98:100	76.6	99:100	129.6 (increased)
23	Opioids	–36.4	96:97	18	95:96	45	98:97	74.8	97:97	35.9	94:96	41.3	100:97	77.7 (increased)
24	Opioids	–16.9	97:97	45.2	99:98	1.49	100:99	3.8	99:100	33.9	99:100	N/A	N/A	50.8 (increased)
26^a^	Opioids	–77.1	84:79	–82.5	92:91	N/A	N/A	N/A	N/A	N/A	N/A	N/A	N/A	5.4
27	Stimulants	–15.2	100:100	–11.6	98:100	10.1	98:100	–27.0	99:97	–25.7	100:98	–1.6	98:100	13.6 (increased)
28	Alcohol	–33.3	99:100	–29.4	99:98	–48.3	99:100	–10.9	99:100	–15.6	99:100	–11.6	100:99	21.8 (increased)
29^a^	Alcohol	–41.4	98:100	–18.0	99:99	20.6	99:99	28.2	99:100	N/A	N/A	N/A	N/A	69.6 (increased)
30	Opioids	1.3	96:94	38.6	98:93	7.9	98:95	11.5	98:91	13	98:98	–8.9	99:98	10.2
31	Opioids	–38.4	88:62	–282.7	96:33:00	–166.4	96:03:00	–190.1	97:03:00	N/A	N/A	N/A	N/A	151.6

^a^Participants did not complete the study, as they left the voluntary program.

^b^N/A: Not available.

^c^There was a holiday during the participant’s stay, and hence, the maximum number of sessions completed was four.

^d^Due to a technical issue, participant 13 was not administered an assessment task following the second intervention, and the participant took another intervention task instead. Attentional bias assessment was performed only after the fourth session.

### Acceptability of the Intervention

Of the 30 participants, 22 (73%) completed the perspective questionnaires. All the participants sampled rated the app as at least very easy (10/22 participants) or extremely easy (11/22 participants) to use, with one participant describing it as “like a primary school application” [Participant 1]. Participants also commented on the adequacy of the provided instructions:

Easy to follow instructions and exercise.

Participant 3

First of all you give me instructions how to press.

Participant 22

Other participants felt that the app was easy to use due to the simplicity of the task:

No need to take time to think, just look at the photos and press either one of them.

Participant 6

Just to follow the asterisk * star.

Participant 15

Just need to follow the stars.

Participant 24

Participants commented on the ease of responding to the task:

Only 2 options – left or right that’s why it is easy.

Participant 4

Just press only.

Participant 10

Participants also felt that the task could be undertaken by a diverse group of participants

But both young and old adults would be able to.

Participant 7

Very simple and easy to be administered to subjects at almost any level of intelligence and age.

Participant 3

With regard to interactivity, eight participants rated the app as extremely interactive, nine rated it as very interactive, and five rated it as moderately interactive. Participants commented that the application was “like playing the game” [Participant 4] and that the app “becomes more engaging” [Participant 3] over time.

With respect to motivation, there was a range of views: Eight participants reported being extremely motivated, four participants reported being very motivated, five participants reported being moderately motivated, four participants reported being slightly motivated, and one participant reported not being at all motivated. Participants who were motivated shared that the app helped them “pass time” [Participant 1] and that it was “just like playing game” [Participant 4]. Other participants were motivated, as they felt that the app “makes me feel better” [Participant 21] and could “help me with my treatment” [Participant 29]. Some participants who indicated that they were motivated in using the app highlighted possible reasons:

Continually using on a daily basis will become repetitive and boring.

Participant 3

It is a repetitive task and some may find it boring to continue using it, unlike a game which really interacts with the user.

Participant 5

If I concentrate, if I do this all the way, boring.

Participant 10

The participant who was not at all motivated commented that he/she finds that the app “doesn’t help with my addiction problem” (Participant 22).

There was a range of responses to the question concerning whether the images reminded the participants of their drug use: 5 participants responded very, 4 responded moderately, 3 responded slightly, and 10 responded not at all. With regard to participants’ confidence in managing their underlying addiction problem, 54% (12 participants) reported no change in their confidence level before and after using the app, and 10 participants reported a change in their confidence level, with 8 participants reporting a positive change.

All participants were invited to provide any additional feedback they had, and these were mainly related to the need to concentrate on the task:

Put your mind on it, follow the star, it would not go wrong.

Participant 14

Must be alert.

Participant 23

It requires your full attention because the switch between the image and the asterisk is very fast so I have to be really focused.

Participant 5

## Discussion

### Principal Findings

The results from our study answered our intended research questions. In the published protocol [23], the study was proposed to be feasible if 25% invited agreed to participate and 60% of the recruited patients adhered to the planned intervention. Our results demonstrated the feasibility of the study in terms of participation and adherence. A 25% recruitment rate was necessary because of the strict inclusion and exclusion criteria. Participation rates are expected to be lower, as individuals with a prior history of withdrawal seizures or any prior history of diagnosed seizures and individuals with moderate to severe psychiatric conditions (as assessed clinically) are excluded. The acceptance rates in our study are higher potentially due to the diversity of substance disorders considered and because most of the participants who sought help were patients with opioid dependence who did not have a prior history of withdrawal seizures, as withdrawal seizures are not common in opioid withdrawal. It is also important to recognize that our inpatient detoxification and rehabilitation program are entirely voluntary, and therefore, individuals are free to request discharge should they not be motivated to stay on. Despite the nature of our program, the adherence rate for this study was not affected. One of the other objectives of the feasibility study was to determine if the mobile attention bias modification intervention could assess and modify attentional biases. We found that the mobile intervention was capable of reducing attentional biases in most of the participants, although there were individuals who did not present with baseline biases.

In the protocol, acceptability is defined as the willingness to use the app daily, if at least 30% of the participants rate the ease of use, interactivity, and motivation positively and if at least 30% of the participants perceived a change in their confidence in managing their addictive disorders after receiving three intervention tasks. Except for one participant who withdrew from the study, the remaining participants were amenable to using the app daily (except for those who decided to leave the ward prematurely and hence did not complete the planned interventions). All these individuals decided to leave prematurely for reasons not related to the study, and there were no adverse outcomes reported during the course of the study. In addition, 100% of the participants rated the app to be either very easy or extremely easy to use, which exceeded our projection of 30%. Moreover, 77% of the participants rated the app as very or extremely interactive and 54% reported being very or extremely motivated to use the app, which exceeded our projection of 30%. Finally, 36% of the participants reported a change in their confidence in managing their addictive disorders, which is congruent with our projection.

In the qualitative feedback, 10 participants reported that the images included did not remind them of their substance use. The images used in the existing app might be different from the images of the substances that they have previously used and thus did not manage to capture their attention. This is in line with a previous commentary [24], which reported that one of the key factors leading to the poor reliability of the visual probe task is that of the nature of the stimulus used. That study [24] highlighted the importance of personalization of the stimulus presented to the participants, as it is postulated that stimulus that is relevant and identifiable to the participant would increase the baseline attentional bias score and provide evidence of greater change in the magnitude of attentional biases. Most of the images included in the existing mobile app were extracted from the internet through the United States Drug Enforcement Agency media library. Some of the images were extracted from Singapore’s Central Narcotics Bureau’s website. It might be possible that the images included do not approximate and are not realistic enough for participants. In our image set for opioids, we showed images of oxycodone and morphine pills, but in Singapore, these are not commonly abused. In addition, in our image set for cannabis, we showed images of spice, which is also not commonly abused in Singapore.

There are clearly several research implications arising from this study. The qualitative feedback from the acceptability part of the study suggests that it is important for us to consider involving patients in improving the app, to allow for personalization of the images and other functionalities of the app. Participatory research methods could be considered, in particular, for focus groups and codesign workshops. In their review, Zhang et al [25] reported that participatory research design methods have been widely applied in both medicine and psychiatry. For psychiatry, these methods have been applied mainly for perinatal depression, dementia, self-harm, and general and youth mental health issues. Their previous review [14] of attention bias and cognitive bias apps in the published literature and the commercial stores revealed that there is a disconnect between academics and developers. Through participatory design, there is potential to enhance the existing app by involving patients and health care professionals in a joint codesign in order to create an app that is more feasible; acceptable; and capable of detecting and modifying biases in opioid, cannabis, stimulants, and alcohol disorders. Our results might also be affected by the images that we chose to include in the app. In the next iteration of this intervention, we will recommend that participants rate the relevance of the images first, before embarking on the actual intervention. Our study also showed that some participants who are in the rehabilitation phase might not present with baseline attentional biases. Thus, this suggests that future research on such an intervention among individuals who are undergoing rehabilitation ought to consider assessing baseline attentional biases first; otherwise, the intervention would be futile in modifying biases.

### Conclusions

To our knowledge, our study is the first study to recruit an Asian cohort of participants with substance use disorders and examine the feasibility and acceptability of the mobile bias intervention. Our results highlight the feasibility to recruit participants to undertake attention bias modification interventions and that participants generally accept a mobile version of such an intervention. Nevertheless, our acceptability data highlight that there could be improvements in the existing app. It is important for future research to take into consideration our findings and adopt a participatory design approach when refining the conventional visual probe task to cater to the needs of the participants.

## Abbreviations

ASI: Addiction Severity Index
NAMS: National Addictions Management Service
SDS: Severity of Drug Dependence Scale
SF: Short Form
